# ECMO support for endoscopic resection of postpneumonectomy critical central airway obstruction

**DOI:** 10.1111/1759-7714.15456

**Published:** 2024-10-29

**Authors:** Alfonso Fiorelli, Marisa De Feo, Michele Torella, Fausto Ferraro, Andrea Bianco, Giuseppe Vicario, Francesca Capasso, Gaetana Messina, Giovanni Natale

**Affiliations:** ^1^ Thoracic Surgery Unit, Department of Translational Medicine University of Campania Luigi Vanvitelli Naples Italy; ^2^ Cardiothoracic Unit, Department of Translational Medicine University of Campania Luigi Vanvitelli Naples Italy; ^3^ Anesthesiology and Intensive Care Unit, Department of Translational Medicine University of Campania Luigi Vanvitelli Naples Italy; ^4^ Pneumology Unit, Department of Translational Medicine University of Campania Luigi Vanvitelli Naples Italy

**Keywords:** ECMO, lung cancer, rigid bronchoscopy

## Abstract

A 73‐year‐old woman was admitted to our hospital with severe respiratory distress due to postpneumonectomy neoplastic central airway obstruction. An emergency recanalization with rigid bronchoscopy (RB) was planned. Controlled and jet ventilation are routinely used to assure ventilation during RB, but the risk of inadequate oxygenation and removal of carbon dioxide was prohibitively high in this case due to the presence of a single lung. The use of venovenous extracorporeal membrane oxygenation was decided by multidisciplinary team to support ventilation during RB. Complete airway recanalization was successfully achieved without any complications. The patient was discharged 2 days later. Pathology revealed metastatic adenocarcinoma, and the patient was reviewed for oncologic treatment.

## Graphical abstract

The tumor almost completely obstructed the upper portion of the trachea. It was resected by rigid bronchoscopy, and extracorporeal membrane oxygenation supported the ventilation during the procedure. The complete recanalization of trachea was obtained.

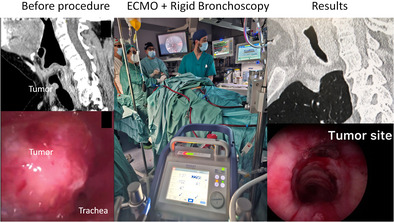

## INTRODUCTION

Central airway obstruction (CAO) is a life‐threatening condition, and rigid bronchoscopy (RB) remains one of the main techniques to relieve obstruction and assure ventilation. Controlled and jet ventilation are routinely used to assure ventilation during RB, but the risk of inadequate oxygenation and removal of carbon dioxide (CO_2_) is extremely high in postpneumonectomy patients with CAO.

Herein, we report the successful endoscopic resection of postpneumonectomy critical CAO with venovenous extracorporeal membrane oxygenation (VV ECMO) support.

## CLINICAL CASE

**FIGURE 1:**
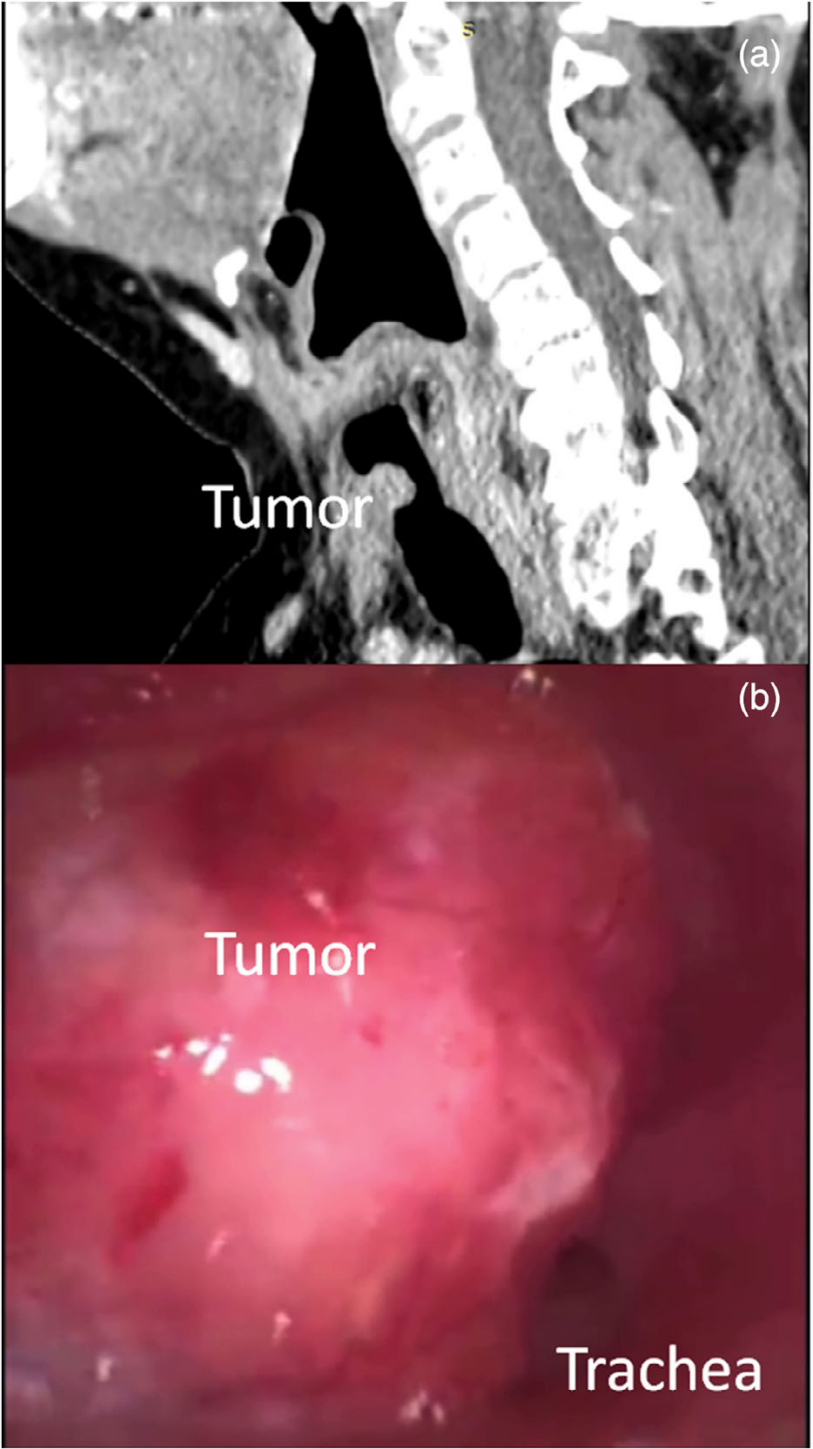
Chest computed tomography (a) and bronchoscopy (b) shows a mass that almost completely obstructs the upper portion of the trachea.

A 73‐year‐old woman was admitted to our hospital with severe respiratory distress. Chest computed tomography (CT) (Figure 1a) and bronchoscopy (Figure 1b) showed a mass almost completely obstructing the upper portion of the trachea. The patient underwent left pneumonectomy 5 years prior for the management of lung adenocarcinoma (T2N0M0) at another hospital, and no adjuvant treatments were administered. After a multidisciplinary consultation, tumor resection through RB with VV ECMO support was planned later on the same day.

**FIGURE 2:**
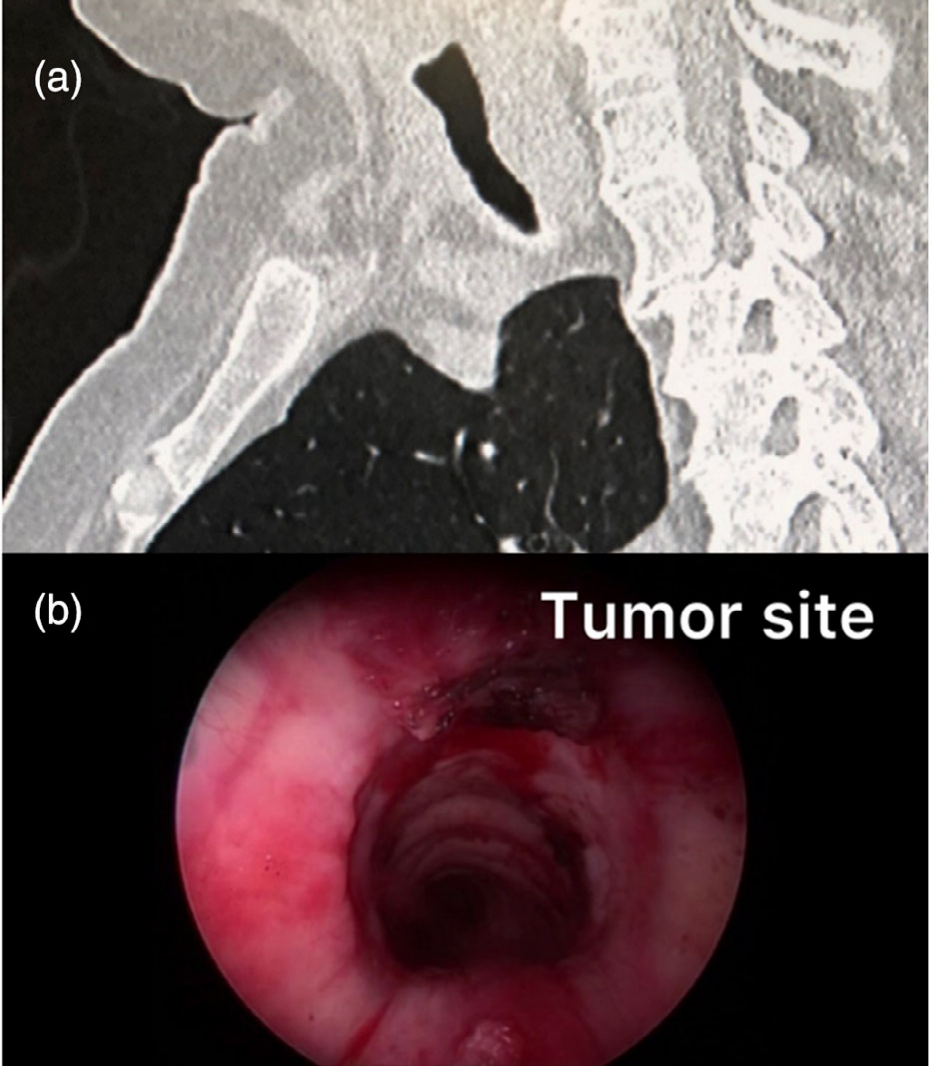
Endoscopy (a) and chest computed tomography (b) shows a recanalization of trachea after the procedure.

The patient was transferred to the operating room. An ECMO cannula was inserted into the right internal jugular and the right femoral vein and intubation performed with an 8.5‐mm RB. The tumor was resected in a standard manner using a rigid barrel, forceps, and neodymium‐doped yttrium aluminum garnet (Nd:YAG) laser. As a complete recanalization of airway was obtained, there was no need of stent insertion (Figure 2b). Subsequently, the patient was weaned from ECMO, reintubated using an 8.0‐mm inner diameter endotracheal tube to protect the only lung in case of sudden bleeding and then transferred to the intensive care unit. She was extubated the next morning and discharged 2 days later. The duration of the procedure was 40 min, while the additional time to place the patient under ECMO was 15 min. A chest CT scan performed 2 weeks later showed normal tracheal patency without tumor recurrence (Figure 1b). Pathology revealed metastatic adenocarcinoma, and the patient was reviewed for oncologic treatments. The entire procedure is summarized in Video S1.

The patient had given a written informed consent for the operation and for the anonymous use of clinical data, photo, and video for scientific purpose only.

## DISCUSSION

The management of this case was challenging. The mass caused near‐total tracheal obstruction resulting in severe respiratory distress requiring urgent RB and tumor resection. Our multidisciplinary team including cardiothoracic surgeons, anesthesiologists, and pneumologists considered this patient too high risk, potentially life‐threatening, for traditional RB. A fraction of inspired oxygen (FiO_2_) of ≤0.4 is recommended to minimize the risk of airway fire related to laser use and is generally well tolerated in patients with healthy lungs.^1^, ^2^ However, this would not have been sufficient to maintain adequate levels of oxygenation and to remove carbon dioxide (CO_2_) in this case, as the presence of a single lung resulted in a reduction of oxygen reserve. In high‐risk patients, some authors^3^, ^4^, ^5^ have proposed the concomitant use of a single lumen tube and RB to allow the ventilation of unaffected lung without increasing the risks of RB complications during resection of endobronchial tumors. However, this strategy was rejected as in this case, the tumor almost completely obstructed the trachea and the presence of the endotracheal tube could interfere with the RB procedures. Finally, VV ECMO was decided as the strategy of choice to support ventilation during RB. VV ECMO has been used to facilitate several RB procedures including tumor resection, stent insertion, and withdrawal of foreign body in high‐risk patients^6^, ^7^, ^8^, ^9^, ^10^; this approach has not been performed previously for the management of postpneumonectomy critical CAO.

VV‐ECMO provided a bridge for ventilatory support and facilitated the procedure by allowing the physician to focus on resecting tumor without concerns regarding oxygenation and CO_2_ removal or other RB complications. As a complete recanalization of airway was obtained, there was no need of stent insertion.^11^ Despite ECMO being useful in our patient's management, it remains an invasive and expensive procedure that requires technical expertise and could be associated with several complications. Thus, it should not be routinely considered as a strategy to deal with a CAO as the majority of cases can be treated successfully without ECMO. Each case should be carefully selected with multidisciplinary discussion to evaluate the risks and benefits of RB with and without ECMO. The use of ECMO as procedural support to RB may prevent avoidable respiratory emergencies and extend these interventions to patients otherwise considered too high risk to treat. However, more data from controlled trials would be required to establish consensus recommendations for the use of ECMO in this setting.

## AUTHOR CONTRIBUTIONS

AF write paper; MT, GV, FC, GM, GN: collection data; MDF, FF, and AB review the paper.

## CONFLICT OF INTEREST STATEMENT

The authors declare no conflicts of interest.

## Supporting information


**Video S1.** The video edited the main steps of the endoscopic resection of the tumor and CT findings before and after treatment.

## Data Availability

Patient had given a written informed consent for the operation and for the anonymous use of clinical data, photo and video for scientific purpose only.
